# False Positive Rate of Rapid Oral Fluid HIV Tests Increases as Kits Near Expiration Date

**DOI:** 10.1371/journal.pone.0008217

**Published:** 2009-12-14

**Authors:** Shelley N. Facente, Teri Dowling, Eric Vittinghoff, Deanna L. Sykes, Grant N. Colfax

**Affiliations:** 1 HIV Prevention Section, San Francisco Department of Public Health, San Francisco, California, United States of America; 2 Division of Biostatistics, University of California San Francisco, San Francisco, California, United States of America; 3 Office of AIDS, Center for Infectious Diseases, California Department of Public Health, Sacramento, California, United States of America; McGill University Health Center, Canada

## Abstract

**Background:**

Because a recent cluster of false positive results on the OraQuick *ADVANCE*® Rapid HIV-1/2 Antibody Test occurred in San Francisco on test kits close to their expiration date, we decided to assess the relationship between time to expiration and rate of false positive results from tests used with oral fluid.

**Methodology/Principal Findings:**

We analyzed results of 20,904 tests with either an initial HIV-negative result (n = 20,828) or a preliminary positive result that was then negative on confirmatory tests (n = 76). We computed specificity for kits with time to expiration from ≤1 to ≥6 months, with exact binomial confidence intervals, then used logistic regression to estimate the independent association of time to expiration with false positive results, adjusting for site and technician effects. For 1,108 kits used in the last month before expiration, specificity was 98.83% (95% exact binomial confidence interval (CI) 98.00%–99.37%); the upper bound is below the claimed specificity of 99.60%. After adjustment using regression standardization for the effects of site, test lot, and technician factors, adjusted specificity in the last month before expiration was 99.18% (95% bootstrap confidence interval 98.60–99.57%).

**Conclusions/Significance:**

We found that specificity of the OraQuick *ADVANCE*® with oral fluid declined significantly with ≤1 month remaining to expiration, leaving little margin for error from other sources.

## Introduction

In June of 2004, the OraQuick *ADVANCE*® Rapid HIV-1/2 Antibody Test (OraSure Technologies, Inc., Bethehem, PA) was approved as a CLIA-waived rapid HIV test for use with oral mucosal transudate (oral fluid) specimens in addition to whole blood.[1] In February of 2005, this test first became available for point-of-care HIV antibody testing in publicly-funded HIV testing sites in San Francisco. Because California regulations required phlebotomy certification before performing a fingerstick or blood draw (California Code of Regulations 17§1034; California Business and Professions Code §1240-1246.5), most community-based testing sites in San Francisco were prevented from performing rapid HIV tests using whole blood specimens, and therefore the advent of an oral fluid option led to a swift, significant increase in rapid testing in public HIV test sites (Facente, presented at HIV Diagnostics: New Developments and Challenges; Orlando, FL, 2005).

United States regulations currently require that all reactive rapid HIV tests be considered “preliminary positive” and confirmed in a high-complexity laboratory via Western blot (WB) or indirect immunofluoresence assay (IFA).[2] In cases where the WB or IFA is negative or indeterminate, the results are considered “discordant” and follow up testing is indicated. This follow up testing is needed to determine whether the original reactive result was a false positive or if the patient has HIV infection but is in early seroconversion, causing the tests used for confirmation – some with lower sensitivity – to fail to detect antibodies.[3] From 2003 through early 2006 in San Francisco, all patients with discordant results were asked to submit blood specimens after one month for follow up testing according to the above protocol. This was done regularly with all patients who were successfully contacted one month after the initial result disclosure. In February of 2006, with the agreement of the California Department of Public Health, Office of AIDS and Centers for Disease Control and Prevention, San Francisco began using a new algorithm that included the use of Nucleic Acid Amplification Testing (NAAT) to resolve most discordant tests from the first visit (Dowling and Facente, presented at XVI International AIDS Conference; Toronto, Canada, 2006). From this point forward, follow up testing was not regularly done if an oral fluid rapid HIV test was reactive but blood drawn that same day yielded non-reactive results for another OraQuick rapid test, an EIA, an IFA and/or WB, and NAAT.

According to the manufacturer, the OraQuick *ADVANCE*® has a specificity of 99.8% (95% Confidence Interval (CI) 99.60%–99.89%) when used with oral fluid.[4] However, clusters of excess false positive results have been well documented[5]–[8], including in San Francisco. To date, these false positive clusters have remained unexplained, with issues such as test kit lot defect, storage or testing area temperature, and operator error being generally ruled out as causes.[9]

In April – July 2008, we noticed a cluster of false positive test results in San Francisco which occurred with kits close to their expiration date, and decided to further investigate the association between kit expiration and false positive results. We hypothesized that there would be a greater probability of false positive results as the kit approached its expiration date.

## Materials and Methods

### Test Kits

The OraQuick *ADVANCE*® test kits used in this analysis were provided at no cost to the San Francisco Department of Public Health from the California Department of Public Health, Office of AIDS, then distributed to HIV testing sites located throughout the city of San Francisco. Test kits were shipped directly from the manufacturer to the San Francisco Department of Public Health upon ordering, and external controls were run on all new boxes of test kits upon opening before use with patients. The shelf life of the test kits used in this analysis was six months, with the exception of a small number of kits that were an early version of the OraQuick *ADVANCE*® that was FDA-approved with a longer shelf life. Actual expiration dates for these kits were changed in our analysis to reflect a consistent 6-month shelf life. Results for 56 kits (including 1 false positive result) used shortly after the adjusted expiration date were omitted from the analysis.

A new version of the OraQuick *ADVANCE*®, which became available to consumers in February 2009, was FDA-approved with a 12-month shelf life following a change in manufacturing and quality control processes to increase consistency of the product[10]. No kits of this enhanced version were used in this analysis.

### Test Records

All HIV antibody tests run in publicly-funded test sites in California are recorded on a standardized laboratory requisition slip. In San Francisco, these slips are submitted on a monthly basis to the San Francisco Department of Public Health for review and data entry. Test technicians at each testing site are trained and certified to run the OraQuick *ADVANCE*®[11] and are specifically instructed to systematically record lot number, expiration date, specimen date, specimen type, and test result for all rapid HIV tests they conduct.[12] Each of these details are individually entered into a database, along with final confirmation results for all positive tests. Extra details and technician notes are maintained for all discordant test results. For each test, we computed time to expiration as the interval from the date the kit was used to the last day of the month and year of the expiration date printed on the test pouch, as recorded in the database.

### Statistics

Specificity was computed for kits for time to expiration by month, from ≤1 to ≥6 months until expiration, with exact binomial confidence intervals. Logistic regression was then used to estimate the association of time to expiration with a false positive result. We tested for trend in the false positive rate across months using an orthogonal contrast; this procedure can be viewed as a linear regression of the month-by-month log odds ratios on the number of months to expiration. [13] In addition, we adjusted for the effects of test site, test lot, and technician. Because there were only 76 false positive results, but numerous lots (n = 129), technicians (n = 210), and sites (n = 22), it was not possible to adjust for these factors by the conventional means of including indicator variables for each lot, technician, and site in the model. To deal with this problem, we classified sites, lots, and technicians according to whether their observed false positive rates were ≤0.5%, >0.5% and <1.0%, or ≥1.0%, representing acceptable, borderline, and clearly unacceptable rates, and then controlled for these classifications. Goodness of fit was assessed using the Hosmer-Lemeshow statistic.

We calculated adjusted specificity by months to expiration using regression standardization[14]. Specifically, the fitted probability of a true negative result was computed for each of the 20,904 observations for all six possible values of months to expiration, holding all other covariates at their observed values. Then specificity for each month was estimated by averaging – over the identical covariate distributions – the 20,904 fitted values for that month. In a final step, bias-corrected bootstrap percentile confidence intervals were calculated for these regression-standardized specificities.

We considered associations with p-values<0.05 to be statistically significant. All analyses were done using Stata Version 11 (Stata Corp., College Station, TX).

## Results

Results were analyzed for 20,904 tests performed between January 2005 and December 2007, with either an initial HIV-negative result (n = 20,828), or a preliminary positive result that proved to be false positive after confirmation by IFA or WB (n = 76). Test results by months remaining until expiration are shown in Table 1. Of 1,108 test kits used with ≤1 month until expiration, 13 (1.17%) were false positive, corresponding to a sample specificity of 98.83% (exact binomial 95% CI 98.00%–99.37%).

**Table 1:** Test specificity by time to expiration.

Time to Expiration (months)	Total Tests (N)	False Positive Tests (N, %)	Specificity (%)	95% Exact Binomial Confidence Interval
≤1	1108	13 (1.17%)	98.83	98.00–99.37
2	2206	14 (0.63%)	99.37	98.94–99.65
3	4968	24 (0.48%)	99.52	99.28–99.69
4	6770	16 (0.24%)	99.76	99.62–99.86
5	4601	8 (0.17%)	99.83	99.66–99.92
≥6	1251	1 (0.08%)	99.92	99.56–99.99+

**Figure 1:**
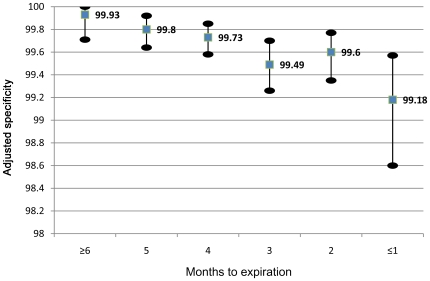
Regression standardized test specificity by month to expiration, adjusting for site, lot, and technician. The blue squares in the figure represent the mean adjusted specificity of the tests, and the black ovals represent the limits of the 95% bootstrap confidence interval.

In the unadjusted logistic regression, the odds of a false positive result were higher for the 1,108 test kits used ≤1 month before expiration, compared to 1,251 kits with ≥6 months until expiration (Odds Ratio (OR) 14.8, 95% CI 1.94–113.6, p = 0.009). False positive rates were also elevated for 2,206 kits with two (OR 7.98, 95% CI 1.05–60.8, p = 0.045) and 4,968 kits with three (OR 6.07, 95% CI 0.82–44.9, p = 0.08) months until expiration. Although the rates for months two and three were similar (OR 1.32, 95% CI 0.68–2.55, p = 0.42), the linear trend across the six months was highly statistically significant (p = 0.0008). Results were similar in the adjusted model, shown in Table 2. The contrast between months one and six (OR 11.5, 95% CI 1.49–89.4, p = 0.019) and the trend across months (p = 0.0057) both remained strong. Six of 129 lots (4.7%), eight of 210 technicians (3.8%) and two of 22 sites (9.1%) had false positive rates of 0.5% to 1.0%, while three lots (2.3%), 13 technicians (6.2%), and two sites (9.1%) had rates greater than 1.0%. These categorizations of lot and technician but not site were important independent predictors in this model, which fit well (Hosmer-Lemeshow p-value = 0.77). Results were similar using other specifications of the adjusted model, and in sensitivity analyses, we found no evidence for effects of calendar month (p = 0.51 for heterogeneity) or calendar year (p = 0.22), after adjusting for lot, technician, and site. In the last month before expiration, adjusted specificity estimated using regression standardization was 99.18% (95% bootstrap confidence interval 98.60–99.57%) (Figure 1).

**Table 2:** Multivariable logistic model for false positive test results.

Predictor		Adjusted Odds-Ratio	95% Confidence Interval	P-value
Time to Expiration (months)	≤1	11.5	1.49–89.4	0.019
	2	5.54	0.71–42.9	0.10
	3	7.05	0.95–52.6	0.06
	4	3.70	0.49–28.2	0.21
	5	2.72	0.34–21.9	0.35
	≥6	*ref*	-	-
Site False Positive Rate (%)	>1.0	0.74	0.36–1.54	0.42
	0.5–1.0	1.86	0.98–3.54	0.06
	<0.5	*ref*	-	-
Technician False Positive Rate (%)	>1.0	21.5	10.6–43.7	<0.001
	0.5–1.0	7.79	3.89–15.6	<0.001
	<0.5	*ref*	-	-
Lot False Positive Rate (%)	>1.0	4.57	2.27–9.21	<0.001
	0.5–1.0	4.77	2.59–8.78	<0.001
	<0.5	*ref*	-	-

## Discussion

We found that the rate of false positive results was significantly higher for oral rapid HIV test kits used with ≤1 month remaining until expiration as compared to kits used with ≥6 months remaining until expiration, after adjustment for the effects of technician and site. Before adjustment, both the point estimate (98.83%) and upper bound of an exact 95% confidence interval (99.37%) for the specificity of tests used with ≤1 month until expiration were below the lower bound for specificity claimed by the manufacturer (99.60%). After regression standardization, the point estimate (99.18%) and upper 95% bootstrap confidence limit (99.57%) both remained below the manufacturer's lower bound. Although residual confounding cannot be ruled out, this suggests that site, lot, and technician effects do not explain our findings, and reflect the rigorous quality assurance system that San Francisco has implemented since the beginning of rapid HIV testing.[15] However, no matter how rigorous a quality assurance system is, some errors will inevitably occur, especially in a non-clinical setting. For example, a well-intentioned technician may swipe the patient's gums with the test kit paddle twice rather than once in an effort to ensure adequate sample collection, or a site's lighting may be improvised in a mobile van, making it more difficult for some technicians to clearly see an extremely faint test line. Ideally, a CLIA-waived test kit will be sufficiently robust to mitigate these factors. It is clear from our results that as these OraQuick *ADVANCE*® test kits age, the overall testing specificity decreases when used with oral fluid, and that tests used within one month of expiration leave little margin for errors from these other sources.

We also found an independent association of test technician on specificity in our adjusted analysis. However, several of the technicians with high false positivity rates had performed very few tests overall, making it difficult to reliably identify those with higher expected long-term rates. Furthermore, test technicians in publicly-funded test sites in the State of California attend a ½ day training, pass a multi-stage proficiency test, and must successfully complete competency assessment testing at least once per year following initial certification. Despite this, they do not have extensive laboratory training, and it is unrealistic to expect that they will always conduct the test perfectly and with no variation. Again, any CLIA-waived, point-of-care test should be sufficiently robust to maintain accuracy given inevitable variations in technician performance.

Since the first cluster of false positive results in San Francisco in late 2005, significant attention has been paid here to a variety of other factors that could possibly be the cause of such a cluster, particularly factors by site and technician. Test storage practices by site, temperature regulation and monitoring at each site, conditions during transport of test kits (i.e. with mobile testing), eyesight of test readers, lighting conditions at each site, and the opportunity for disruption of test kits during processing were all factors that were reviewed at length at all sites in San Francisco. No patterns related to false positive results were detected and no dramatic variations between sites were observed, leading us to believe these factors were not significant to this analysis.

Previous investigations have not successfully determined a cause for the intermittent clusters of false positive results seen around the country since the introduction of the OraQuick *ADVANCE®* when used with oral fluid.[5]–[9] Our results, however, offer one potential explanation. They also fit with patterns of test kit use relative to the expiration date, as a result of shipping schedules and quantity of test kits in stock for a particular jurisdiction.[16]

Rapid HIV testing is a critical public health intervention. Not only does it improve access to serological testing for millions of Americans at risk for HIV, but it does so while providing same-day screening results to all patients, with only people testing preliminary positive needing to wait the 7- to 14-days for IFA or WB results. In the United States, the Centers for Disease Control and Prevention as well as numerous state and city governments have spent millions of dollars purchasing the OraQuick *ADVANCE*® as a cornerstone of this intervention.[17]–[19] This test, used with oral fluid, is currently the most widely used point-of-care rapid HIV test in the country (Wesolowski, Burstein, Zhu, and Ethridge. Presented at HIV Diagnostics: New Developments and Challenges; Orlando, FL, 2005). For these reasons, it is imperative to resolve issues of clustering false positive results with oral fluid in order to continue its use to assist in diagnosing new HIV infections.

Anecdotal experiences from various jurisdictions indicate a marked reduction in false positive results using the most recent version of the OraQuick *ADVANCE*®, released in February 2009. However, additional analyses should be conducted with data sets in jurisdictions using these new tests, to confirm that test remains robust throughout the current 12-month shelf life claim when the test is run in typical field conditions, and that policy changes and procedural adaptations – such as discontinuing use when kits are within a month of expiration – are not necessary in order to ensure adequate specificity. Finally, CDC and Association of Public Health Laboratories' approval of a rapid multi-test algorithm for point-of-care resolution of false positive results in the United States could also address these issues and eliminate the need, in most cases, for additional testing and prolonged uncertainty regarding an individual's HIV status.
